# The Reliability and Validity of Recalled Body Shape and the Responsiveness of Obesity Classification Based on Recalled Body Shape Among the Chinese Rural Population

**DOI:** 10.3389/fpubh.2022.792394

**Published:** 2022-05-03

**Authors:** Wei Liao, Xiaotian Liu, Ning Kang, Miaomiao Niu, Yu Song, Lulu Wang, Dandan Wei, Pengling Liu, Chunyang Sun, Zhenxing Mao, Jian Hou, Chongjian Wang, Yuqian Li

**Affiliations:** ^1^Department of Epidemiology and Biostatistics, College of Public Health, Zhengzhou University, Zhengzhou, China; ^2^Department of Occupational and Environmental Health Sciences, College of Public Health, Zhengzhou University, Zhengzhou, China; ^3^Department of Preventive Medicine, School of Medicine, Henan University of Chinese Medicine, Zhengzhou, China; ^4^Department of Clinical Pharmacology, School of Pharmaceutical Science, Zhengzhou University, Zhengzhou, China

**Keywords:** reliability, validity, responsiveness, recalled body shape, rural area

## Abstract

**Background:**

The reliability and validity of recalled body shape were unknown in China. This study was conducted to examine the reliability and validity of recalled body shape as well as the responsiveness of obesity classification by recalled body shape among the Chinese rural population.

**Methods:**

A total of 166 people from the Henan rural cohort were enrolled. The Spearman's correlation coefficient (SCC), intraclass correlation coefficient (ICC), and Cronbach's α were calculated to assess the reliability and validity of recalled body shape. Additionally, the receiver operator characteristic curve (ROC) was performed to assess the responsiveness.

**Results:**

The SCC between the twice recalled body shape ranged from 0.383 to 0.578, and the ICC ranged from 0.357 to 0.615. Besides, the Cronbach's α of the recalled body shape questionnaire was 0.845. At the age of 20–70, the SCC between recalled body shape and actual body mass index (BMI) and waist circumference (WC) ranged from 0.563 to 0.699 and 0.409 to 0.661, respectively. Furthermore, above the age of 20, the area under the curve (AUC) of classifying general obesity and abdominal obesity by recalled body shape ranged from 0.833 to 0.960 and 0.686 to 0.870, respectively.

**Conclusion:**

The results indicated that recalled body shape had moderate reliability, validity, and discriminative degree for earlier obesity among the Chinese rural population.

## Introduction

In recent decades, the disease burden has shifted from primarily infectious to non-communicable chronic diseases (NCDs) globally and in China (1, 2). NCDs have a long latency, which means the exposure to etiological factors may have occurred in the distant past before diseases are diagnosed (3, 4). Therefore, the past exposure to risk factors of NCDs and long-term trends of the exposure may be more significant than the current exposure to risk factors of NCDs. For instance, previous studies found that obesity in childhood and adolescence was associated with many NCDs in adulthood (5–8). However, in many epidemiological studies, the participants' recollection may be the only way to acquire the early exposure and was frequently used (9–11).

The validity of recalled weight at an earlier age has been demonstrated in populations with high education levels (12, 13), but it was affected by elapsed time, current body mass index (BMI), weight gain and loss, and weight variability (14). Nevertheless, among the rural population with low education levels and limited resources, recalling weight may not be accurate because they were less likely to measure their weight and pay less attention to their weight. Thus, recalling body shape may be an effective way to obtain information about previous obesity in the rural population.

The pictorial body diagrams were developed first by Stunkard et al. (15), which was validated by Must et al. (16) in Boston. The results indicated that recalled body shape could provide useful information independent of current weight status. Two other studies conducted in America found that young women can accurately recall their body shape at menarche and indicated that recalled body shape may offer advantages in certain situations (17, 18). Although the pictorial body diagrams have been developed for a long time, the reliability, and validity of recalled body shape were still unknown among a Chinese population. Previous studies in west countries found that body shape trajectories were associated with the incidence of depression, hypertension, type 2 diabetes mellitus (T2DM), cardio-metabolic disease, cancer, and mortality (19–24). As far as we know, relevant research has not been reported among a Chinese population. Currently, participants in a Henan rural cohort have provided body shape information recalled for earlier ages. However, the reliability and validity of recalled body shape as an indicator of earlier obesity were still unknown. Thus, the aims of this study were to examine the reliability and validity of recalled body shape as well as the discriminative degree of obesity classification *via* recalled body shape among the Chinese rural population.

## Materials and Methods

### Study Population

The population of this study was selected from the Henan rural cohort, a large population-based prospective cohort study aiming to explore the prevalence and incidence of chronic diseases and their risk factors. In brief, the cohort study recruited 39,259 participants aged from 18 to 79 living in Yuzhou, Suiping, Tongxu, Xinxiang, and Yima counties of Henan province in China *via* multi-stage stratified cluster sampling. From 2015 to 2017, the baseline survey of this cohort has been completed with a response rate of 93.7%. The detailed information of the cohort has been described in a previous publication (25).

The recruitment for the reliability and validity of recalled body shape examined in the current study was conducted from September to November 2020. A total of 166 people were willing to participate in this study, whose previous height, weight, and waist circumference were recorded at local medical facilities. To test the reliability of the recalled body shape, participants took the second recalled body shape questionnaire survey about 4 weeks after completing the first. In the second recalled body shape questionnaire survey, a total of 155 participants were surveyed again with a response rate of 93.37%, while 11 participants lost to follow-up or refused to be investigated. Most specialists believe that the time interval of a repeat survey of 4 weeks was rational, which can avoid the influence of the first survey (26, 27).

The Henan Rural Cohort Study was approved by the Zhengzhou University Life Science Ethics Committee and conducted in accordance with the principles of the Declaration of Helsinki [Code: [2015] MEC (S128)]. Participants were required to provide informed consent, and both the researchers and respondents agreed to use the data for scientific research purposes only.

### Data Collection

According to a face-to-face interview, a structured questionnaire was asked by well-trained research staff. We collected participants' demographic characteristics, lifestyle factors and recalled body shapes at different ages. Demographic characteristics included age in years, gender, marital status (married/cohabiting and widowed/separated/divorced/single), education level (elementary school or below, junior high school, and senior high school or above), and average monthly income (<500 RMB, 500- RMB, and ≥1,000 RMB). Lifestyle factors including smoking and drinking (never, former, and current) were also collected. The definitions of current smoking and drinking can be seen in a previous publication (28).

The pictorial body diagrams were developed first by Stunkard et al. (15), which is shown in Supplementary Figure 1. Participants were asked to report which one of the 9 pictorial body diagrams best reflected their body shape at the age of 5, 10, 20, 30, 40, 50, 60, 70, and current. Four weeks after the first recalled body shape survey, the second recalled body shape survey was conducted for the reliability test. The previous height, weight, and waist circumference (WC) of participants were obtained from physical examination data at local medical facilities. The current height and weight of participants were measured twice, and the average readings were computed to analyze. The body mass index (BMI, kg/m^2^) was calculated through weight (kg) divided by square of height (m).

### Gold Standard Definition of Obesity

At the age of 5, the gold standard of definition of obesity was height-for-weight according to the reference standards for the growth and development of children under 7 years old in China developed in 2009 (29). At the age of 10, the gold standard of definition of general obesity was according to BMI threshold for obesity in children aged 7–18 years established by the Working Group of Obesity in China (WGOC) in 2004 (30), while abdominal obesity was defined by the WC threshold established by WGOC in 2010 (31). For the age at 20 or above, in accordance with the Chinese standard of BMI and WC (32), BMI ≥ 28 kg/m^2^ was defined as general obesity, and WC ≥ 90 cm for men and WC ≥ 80 cm for women were classified as abdominal obesity. In addition, we also performed a sensitivity analysis using the World Health Organization's (WHO) definition of obesity. At the ages of 5 and 10, the gold standard of the definition of obesity is that BMI-for-age is >2 standard deviations above the WHO Growth Reference median (33). For the age of 20 or above, in accordance with the WHO standard of BMI and WC (33, 34), BMI ≥ 30 kg/m^2^ was defined as general obesity, and WC ≥ 102 cm for men and WC ≥ 88 cm for women were classified as abdominal obesity.

### Statistical Analysis

Statistical descriptions of continuous and categorical variables were presented as mean with standard deviation (SD) and frequency with percentages, respectively. *T*-test was performed to compare differences between different groups for continuous variables, while the Chi-squared test was utilized for categorical variables.

In order to evaluate the consistency between the twice recalled body shape, the scatter plot of first vs. second recalled body shape at different ages was drawn. It was considered as a good consistency that the difference between the twice recalled body shape was no more than 1. To assess the test-retest reliability of recalled body shape, the Spearman's correlation coefficient (SCC), the Pearson correlation coefficient (PCC^a^), and the intraclass correlation coefficient (ICC) between the twice recalled body shape were calculated. Besides, Cronbach's α was calculated to evaluate the internal consistency. The SCC between the first recalled body shape and the actual BMI/WC/weight was calculated to assess the validity of recalled body shape. Considering the current BMI and age influence on recalled body shape, the partial correlation coefficient (PPC^b^) adjusted current BMI and age was also calculated.

Besides, the receiver operator characteristic curve (ROC) was performed to assess the responsiveness of obesity classification based on recalled body shape at different ages and to find the best cutoff value. The gold standard definition of obesity was described above. The sensitivity and specificity were calculated to measure the accuracy of defining obesity *via* recalled body shape, while the area under the curve (AUC) was utilized to assess the discriminative degree of obesity defined by recalled body shape.

The figures were produced using the R language software 4.0.2. Statistical analyses were performed by SPSS 21.0 software package (SPSS Institute, Chicago), and all *P*-values were two-tailed with a statistical significance level of 0.05.

## Results

### Characteristics of Participants

The characteristics of all 166 participants in this study according to gender are shown in Table 1. Compared with women, men were more likely to have higher education levels and lower average monthly income, and be current smokers and drinkers (all *P* < 0.05). There were no significant differences between men and women in terms of age and marital status (all *P* > 0.05). The age distribution of the study participants according to gender is presented in Supplementary Figure 2. In total, participants aged from 20 to 86. Among both men and women, most participants aged from 50 to 70. The comparison of the characteristics of participants between the current study and the cohort is presented in Supplementary Table 1. Compared with the participants in the cohort, participants in the current study were more likely to be old, men, current smokers, and have a higher level of average monthly income, and were less likely to be current drinkers (all *P* < 0.05). Additionally, there were no significant differences between participants in the current study and the cohort in terms of marital status and education level (all *P* > 0.05).

**Table 1:** The characteristics of participants in this study.

**Characteristics**	**Men (*n* = 89)**	**Women (*n* = 77)**	***P***
Age (year), (mean and SD)	60.45 (12.41)	56.89 (14.27)	0.087
**Marital status** ***n*** **(%)**			
Married/cohabiting	78 (87.64)	71 (92.21)	0.333
Widowed/separated/divorced	11 (12.36)	6 (7.79)	
**Educational level** ***n*** **(%)**			
Elementary school or below	22 (24.72)	39 (50.65)	0.002
Junior high school	47 (52.81)	30 (38.96)	
Senior high school or above	20 (22.47)	8 (10.39)	
**Average monthly income** ***n*** **(%)**			
<500 RMB	25 (28.09)	10 (12.99)	0.032
500- RMB	19 (21.35)	26 (33.77)	
≥1,000 RMB	45 (50.56)	41 (53.25)	
**Smoking status** ***n*** **(%)**			
Current smoking	53 (59.55)	0 (0.00)	<0.001
Former smoking	12 (13.48)	0 (0.00)	
Never smoking	24 (26.97)	77 (100.00)	
**Drinking status** ***n*** **(%)**			
Current drinking	38 (42.70)	2 (2.60)	<0.001
Former drinking	17 (19.10)	0 (0.00)	
Never drinking	34 (38.20)	75 (97.40)	

*SD, standard deviation; RMB, Renminbi*.

*T-test was performed to compare the differences in continuous variables; Chi-square test was used to compare the differences in the categorical variable*.

### Reliability of Recalled Body Shape

**Figure 1:**
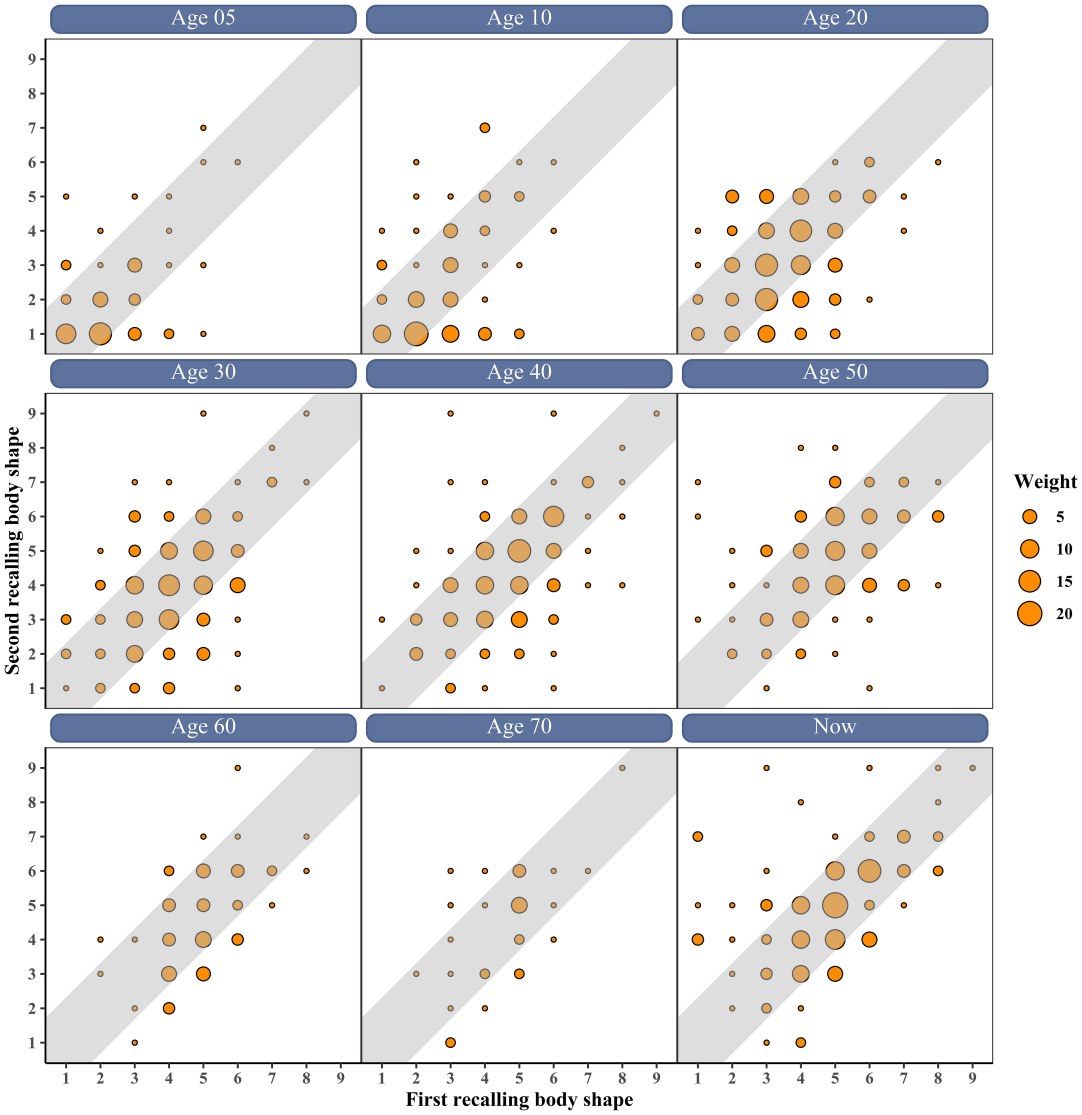
The scatter plot of first vs. second recalled body shape at different ages.

Figure 1 shows the scatter plot of first vs. second recalled body shape at different ages. The results showed that most participants recalled similar body shape twice, with a difference of less than one body shape. Before the age of 40, most participants selected the small body diagrams, while more people selected the larger body diagrams after 40. The correlation coefficients between the twice administration of recalled body shape are shown in Table 2. The mean of SCC was 0.473 between the twice recalled body shape, ranging from 0.383 (age 20) to 0.578 (age 60). After adjusting the current BMI and age, the PCC^b^ between the twice recalled body shape ranged from 0.405 (age 20) to 0.522 (age 5) except for age 50 (0.278; all *P* < 0.05). The mean of PCC ^a^ between the twice recalled body shape was 0.502, ranging from 0.375 (age 50) to 0.640 (age 70). In addition, the mean of ICC between the twice recalled body shape was 0.493, ranging from 0.357 (age 50) to 0.615 (age 70; all *P* < 0.05), Additionally, the Cronbach's α of the recalled body shape questionnaire was 0.845. The correlation coefficients between the twice recalled body shape according to gender are presented in Supplementary Table 2. The SCC between the twice recalled body shape ranged from 0.376 (age 5) to 0.668 (age 60) in men and ranged from 0.338 (age 30) to 0.588 (age 70) in women.

**Table 2:** The correlation coefficients of twice recalled body shape.

**Age**	***N* ^#^**	**SCC**	**PCC^a^**	**PCC^b^**	**ICC**
5	64	0.412*	0.542^**^	0.522^**^	0.533^**^
10	91	0.424^**^	0.470^**^	0.428^**^	0.450^**^
20	155	0.383^**^	0.424^**^	0.405^**^	0.424^**^
30	148	0.428^**^	0.481^**^	0.438^**^	0.474^**^
40	142	0.524^**^	0.532^**^	0.441^**^	0.529^**^
50	118	0.414^**^	0.375^**^	0.278*	0.375^**^
60	62	0.578^**^	0.569^**^	0.462^**^	0.554^**^
70	33	0.541^*^	0.640^**^	0.492^*^	0.615^**^
Now	148	0.557^**^	0.487^**^		0.487^**^

*SCC, Spearmen correlation coefficient; PCC*

a *, Pearson correlation coefficient; PCC*

b *, partial correlation coefficient; ICC, intraclass correlation coefficient*.

# *Sample sizes vary due to miss value*.

* *P <0.05*,

** *P <0.001*.

### Validity of Recalled Body Shape

Table 3 summarizes the correlation coefficients between the first recalled body shape and actual BMI and WC. At the age of 5, the SCC and PCC^b^ between recalled body shape and actual weight were 0.271 and 0.310, respectively. At the age of 10, the correlation between recalled body shape and actual BMI was weak but increased after adjusting for current BMI and age. However, there was a non-statistically significant correlation between recalled body shape and actual WC at the age of 5. At the age of 20–70, the SCC between recalled body shape and actual BMI/WC ranged from 0.563 (age 50) to 0.699 (age 40) and 0.409 (age 20) to 0.661 (age 70), respectively (all *P* < 0.001). The SCC between body shape and BMI/WC at current were 0.669 and 0.622, respectively. Adjusting for current BMI and age had little influence on the correlation coefficients at age 20–40, but the correlation coefficients decreased significantly after age 40. The correlation coefficients between the first recalled body shape and BMI/WC according to gender are summarized in Supplementary Table 3. The SCC between the recalled body shape and weight/BMI ranged from 0.242 (age 5) to 0.734 (age 40) in men and ranged from 0.209 (age 10) to 0.850 (age 70) in women. In addition, the SCC between the recalled body shape and WC ranged from 0.300 (age 10) to 0.680 (age 60) in men and ranged from 0.360 (age 10) to 0.765 (age 70) in women.

**Table 3:** The correlation coefficients between first recalled body shape and BMI and WC.

**Age group**	**BMI (kg/m** ^**2**^ **)/Weight (kg)**		**WC (cm)**		**SCC^†^**	**SCC^*rm*^^‡^**	**PCC^b^**	**PCC^b^^*rm*^**
	***n* ^#^**	**mean (SD)**	***n* ^#^**	**mean (SD)**				
Age 05^a^	88	22.45 (7.46)			0.271^*^		0.310^*^	
Age 10	118	21.51 (5.56)	45	62.04 (7.89)	0.249^*^	0.140	0.314^*^	0.293
Age 20	166	21.07 (3.20)	161	76.06 (9.63)	0.648^**^	0.409^**^	0.610^**^	0.434^**^
Age 30	158	22.59 (2.74)	157	80.18 (10.34)	0.652^**^	0.497^**^	0.627^**^	0.465^**^
Age 40	148	23.44 (3.00)	147	82.98 (10.97)	0.699^**^	0.576^**^	0.567^**^	0.501^**^
Age 50	120	23.83 (3.10)	115	84.93 (9.98)	0.563^**^	0.499^**^	0.351^**^	0.337^**^
Age 60	67	23.65 (3.32)	68	84.79 (12.30)	0.641^**^	0.639^**^	0.392^*^	0.355^*^
Age 70	36	22.93 (3.28)	36	84.34 (10.80)	0.593^**^	0.661^**^	0.176	0.400^*^
Now	166	23.79 (3.27)	166	76.06 (9.63)	0.669^**^	0.622^**^		

*BMI, body mass index; WC, waist circumference; SD, standard deviation; SCC, spearmen correlation coefficient; PCC*

b *, partial correlation coefficient*.

a *At age 05, weight (kg) was used to calculate the correlation coefficient*.

# *Sample sizes vary due to miss value*.

† *Body shape and BMI*.

‡ *Body shape and WC*.

* *P <0.05*,

** *P <0.001*.

### Responsiveness of Defining Obesity by Recalled Body Shape

**Figure 2:**
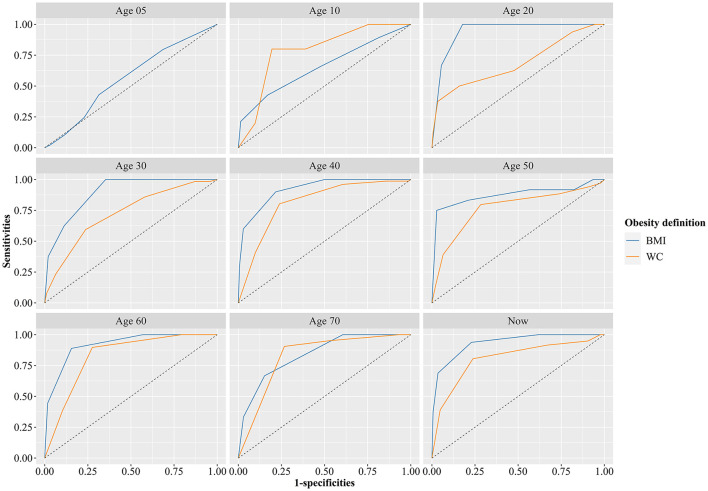
Receiver operator characteristic curve of determining obesity by recall of body shape at different ages (Gold standard definition of obesity at age 5 was weight-for-height based on Chinese standard in 2009, gold standard definition of obesity at age 10 was BMI and WC classification criteria for screening Chinese school-age children developed by the China Working Group on Obesity).

Figure 2 presents the receiver operator characteristic curve of determining obesity by recalled body shape at different ages. The responsiveness of defining obesity by recalled body shape was low at the age of 5. In addition, the responsiveness of defining abdominal obesity by recalled body shape was higher than general obesity at the age of 10, while the responsiveness of defining general obesity by recalled body shape was higher than abdominal obesity after the age of 20. Table 4 shows the best cut-off value to define obesity based on recalled body shape, and the sensitivity and specificity of the definition. After the age of 20, the AUC of defining general obesity by recalled body shape ranged from 0.833 (age 70) to 0.960 (age 20), and the AUC of defining abdominal obesity by recalled body shape ranged from 0.686 (age 20) to 0.870 (age 70; all *P* < 0.05). The results indicated that the responsiveness of defining general and abdominal obesity by recalled body shape was effective after the age of 20. More details can be seen in Table 4. The best cut-off value to define obesity based on recalled body shape, and the sensitivity and specificity of the World Health Organization's definition is presented in Supplementary Table 4. Compared with the Chinese definition of obesity, the best cut-off values of the WHO standard were generally larger.

**Table 4:** The best cut-off value to define obesity based on recalled body shape, and the sensitivity and specificity of the definition.

	**Cutoff value**	**Sensitivity (%)**	**Specificity (%)**	**AUC (95% CI)**	***P***
**BMI**					
**Age group**					
Age 05^a^	Body shape ≥ 3	42.90	68.60	0.564 (0.437, 0.691)	0.320
Age 10^b^	Body shape ≥ 4	42.55	83.10	0.654 (0.550, 0.758)	0.005
Age 20	Body shape ≥ 5	100.00	82.21	0.960 (0.891, 1.000)	0.006
Age 30	Body shape ≥ 5	100.00	64.67	0.895 (0.811, 0.978)	<0.001
Age 40	Body shape ≥ 6	90.00	78.30	0.921 (0.846, 0.995)	<0.001
Age 50	Body shape ≥ 6	83.33	78.70	0.869 (0.709, 1.000)	<0.001
Age 60	Body shape ≥ 6	88.90	84.50	0.920 (0.833, 1.000)	<0.001
Age 70	Body shape ≥ 6	66.70	84.80	0.833 (0.620, 1.000)	0.003
Now	Body shape ≥ 6	93.80	77.10	0.933 (0.874, 0.992)	<0.001
**WC**					
**Age group**					
Age 05^a^					
Age 10^b^	Body shape ≥ 4	80.00	80.00	0.780 (0.586, 0.974)	0.043
Age 20	Body shape ≥ 5	50.00	84.10	0.686 (0.529, 0.844)	0.015
Age 30	Body shape ≥ 5	59.38	76.30	0.725 (0.645, 0.805)	<0.001
Age 40	Body shape ≥ 5	80.30	76.10	0.820 (0.751, 0.889)	<0.001
Age 50	Body shape ≥ 5	79.71	71.70	0.769 (0.681, 0.858)	<0.001
Age 60	Body shape ≥ 5	89.74	72.41	0.835 (0.733, 0.936)	<0.001
Age 70	Body shape ≥ 5	90.48	73.33	0.870 (0.751, 0.989)	<0.001
Now	Body shape ≥ 5	80.50	76.20	0.808 (0.736, 0.881)	<0.001

*BMI, body mass index; WC, waist circumference; AUC, area under the curve; CI, confidence interval*.

a *Gold standard definition of obesity at age 5 was weight-for-height based on Chinese standard in 2009*.

b *Gold standard definition of obesity at age 10 was BMI and WC classification criteria for screening Chinese school-age children developed by the China Working Group on Obesity*.

## Discussion

The results of this study showed that the recalled body shape questionnaire had good internal consistency indicated by Cronbach's α (0.845) above the conventional threshold of 0.700. Besides, the correlation coefficients between twice-recalled body shape, and between recalled body shape and actual BMI/WC were moderate, indicating that recalled body shape can contribute useful information about earlier obesity. Moreover, the high AUC manifested that the responsiveness of classifying general and abdominal obesity by recalled body shape was effective.

In this study, the consistency between the twice recalled body shape was examined, indicating the consistency was moderate. A moderate correlation between recalled body shape and actual BMI/WC were also found, which was similar to the previous study conducted in Harvard (16). Another study in America found that the correlation coefficient between actual BMI and recalled body shape at the age of menarche was 0.61 (17), similar to the current study. However, a previous study conducted in America found that the correlation coefficient between actual BMI and recalled body shape at the age of menarche was 0.77 (18), which was higher than the current study. This may be explained by the age of the participants in this study ranging from 14 to 19.5. The study participants were younger than the participants in the current study and had a short distance from the time point of recall. Additionally, menarche was a particular time, and participants may find it easier to recall the information at that time.

The current study found that the correlation coefficients between recalled body shape and actual BMI/WC/weight were low at the age of 5 and 10, as well as the low AUC of classifying obesity. This may be explained by the time gaps being too long for participants to accurately recall body shape at that time. Another possible reason was that the body images depict adult body shapes, which makes it difficult for participants to recall their childhood body shape based on these images accurately. The current study also found that adjusting for current BMI and age had little effect on the correlation coefficients at the age of 20–40, but the correlation coefficients decreased significantly after the age of 40. The result indicated that the current BMI and age would affect participants' recollections of their body shape in the recent past, but not their recollections of body shape in the distant past, which was consistent with the previous study (16). After the age of 20, the AUC of classifying general obesity ranged from 0.833 to 0.960, and the AUC of defining abdominal obesity ranged from 0.686 to 0.870 (all *P* < 0.05). This result showed that recalled body shape was a good indicator of earlier obesity. Moreover, the current study found that the responsiveness of defining general obesity by recalled body shape was higher than abdominal obesity after the age of 20. This may be due to the images reflecting the whole body so that participants were more accurate in recalling the condition of general obesity.

To the best of our knowledge, this was the first study to examine the reliability and validity of recalled body shape and the responsiveness of obesity classification based on recalled body shape in the Chinese rural population. The results showed that the reliability and validity of recalled body shape were moderate and indicated that recalled body shape could provide useful information about obesity at an earlier age. However, there were also several limitations in this study. Firstly, selection bias may influence the results of this study because reasons for refusing or losing follow-up may be related to the accuracy of recalled body shape. Participants probably refused to be surveyed because they could not accurately recall their previous body shape. Secondly, the previous height, weight, and waist circumference of participants were obtained from physical examination data at local medical facilities, and the actual values of BMI for age 0 and 10 were obtained from relatively young participants, which may lead to bias. Thirdly, the sample size of this study is limited and it is necessary to carry out a study with a larger sample size. Fourthly, the results were based on only one province of China, which might not be a representative sample of the Chinese rural population. However, the rural population of the Henan province accounts for 8.9% of the rural Chinese population, and the results based on this relatively large rural cohort study, to some extent, could represent the Chinese rural population. Moreover, although the correlation coefficients between the twice recalled body shape as well as between recalled body shape and BMI/WC remained significant after adjusting current BMI and age, we did not consider other factors affecting recalled body shape, such as weight gain and loss.

In conclusion, although the validity of recalled weight has been demonstrated in populations with high education levels (12, 14), recalled body shape was an effective way to obtain information about earlier obesity in rural populations with low education levels and limited resources. In the current study, the moderate all over correlation coefficients and high AUC of obesity classification based on recalled body shape suggested that recalled body shape can provide useful information about earlier obesity and have a good discriminative degree for obesity among a Chinese rural population. This evidence may inform life-course epidemiological studies that consider the recalled body shape to indicate earlier obesity.

## Data Availability Statement

The raw data supporting the conclusions of this article will be made available by the authors, without undue reservation.

## Ethics Statement

Written informed consent was obtained from the individual(s) for the publication of any potentially identifiable images or data included in this article.

## Author Contributions

WL: investigation, formal analysis, validation, visualization, writing—original draft, and writing—review and editing. XL: investigation, formal analysis, and writing—review and editing. NK: investigation, validation, visualization, and writing—review and editing. MN: investigation, validation, and writing—review and editing. YS, LW, and DW: investigation and writing—review and editing. PL, JH, and CW: formal analysis and writing—review and editing. CS and ZM: data curation and writing—review and editing. YL: conceptualization, data curation, methodology, and writing—review and editing. All authors critically revised the manuscript and approved the final version for publication.

## Funding

This research was supported by the Foundation of National Key Program of Research and Development of China (Grant No: 2016YFC0900803), the Science and Technology Innovation Team Support Plan of Colleges and Universities in Henan Province (Grant No: 21IRTSTHN029), Key Research Program of Colleges and Universities in Henan Province (Grant No: 21A330007), Foundation of Medical Science and Technology of Henan province (Grant Nos: 201702367 and 2017T02098), and Discipline Key Research and Development Program of Zhengzhou University (Grant Nos: XKZDQY202008 and XKZDQY202002). The funders had no role in the design, analysis or writing of this article.

## Conflict of Interest

The authors declare that the research was conducted in the absence of any commercial or financial relationships that could be construed as a potential conflict of interest.

## Publisher's Note

All claims expressed in this article are solely those of the authors and do not necessarily represent those of their affiliated organizations, or those of the publisher, the editors and the reviewers. Any product that may be evaluated in this article, or claim that may be made by its manufacturer, is not guaranteed or endorsed by the publisher.
