# Supplementary material for: The Reliability and Validity of Recalled Body Shape and the Responsiveness of Obesity Classification Based on Recalled Body Shape Among the Chinese Rural Population
Source: Front Public Health. 2022 May 3;10:792394. doi: 10.3389/fpubh.2022.792394 (PMC9110696; doi:10.3389/fpubh.2022.792394)
Supplement: Supplementary file 1 [file Data_Sheet_1.docx]

**
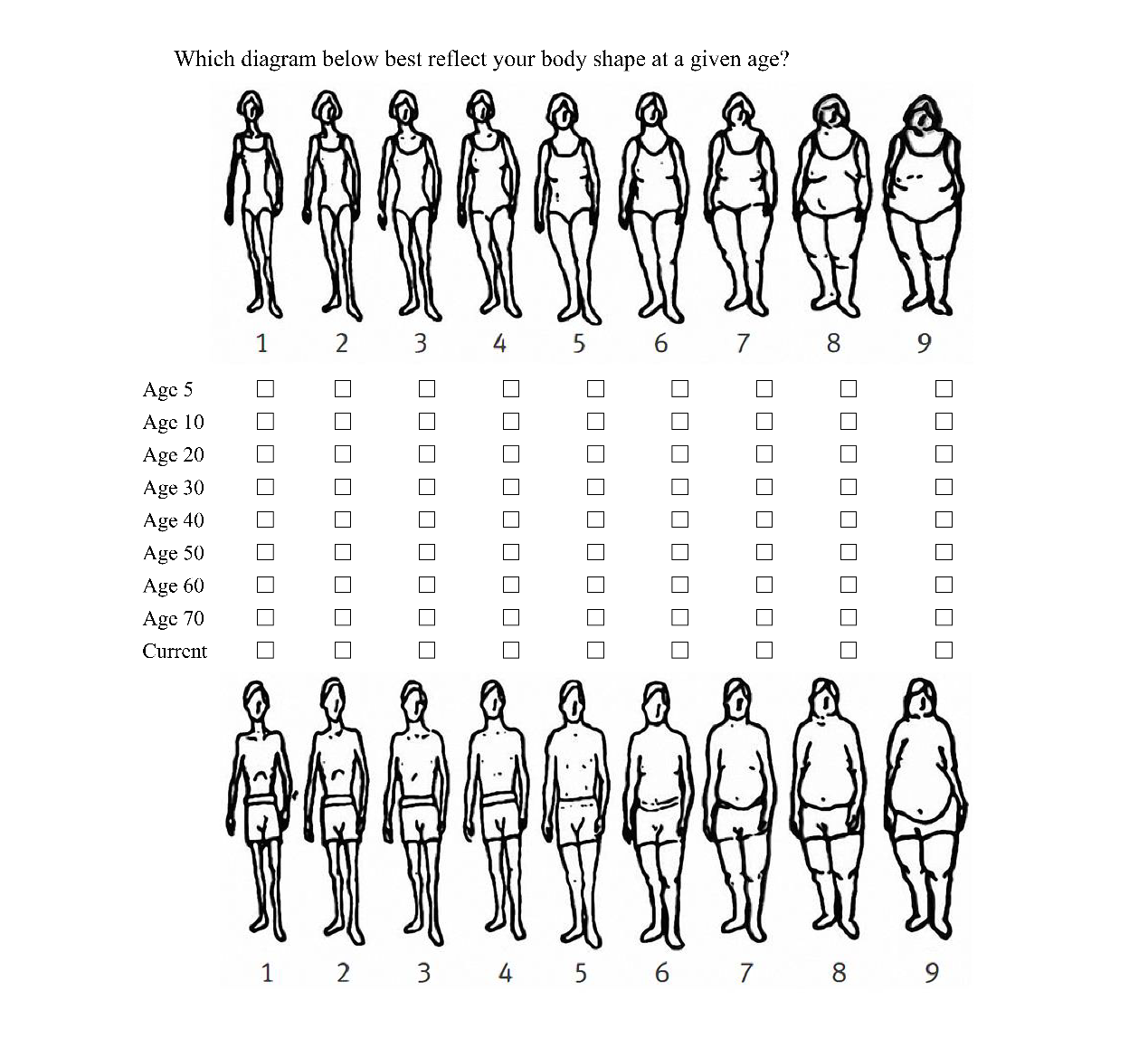
**

**Supplementary figure 1.** Somatotype drawings for recall.

**
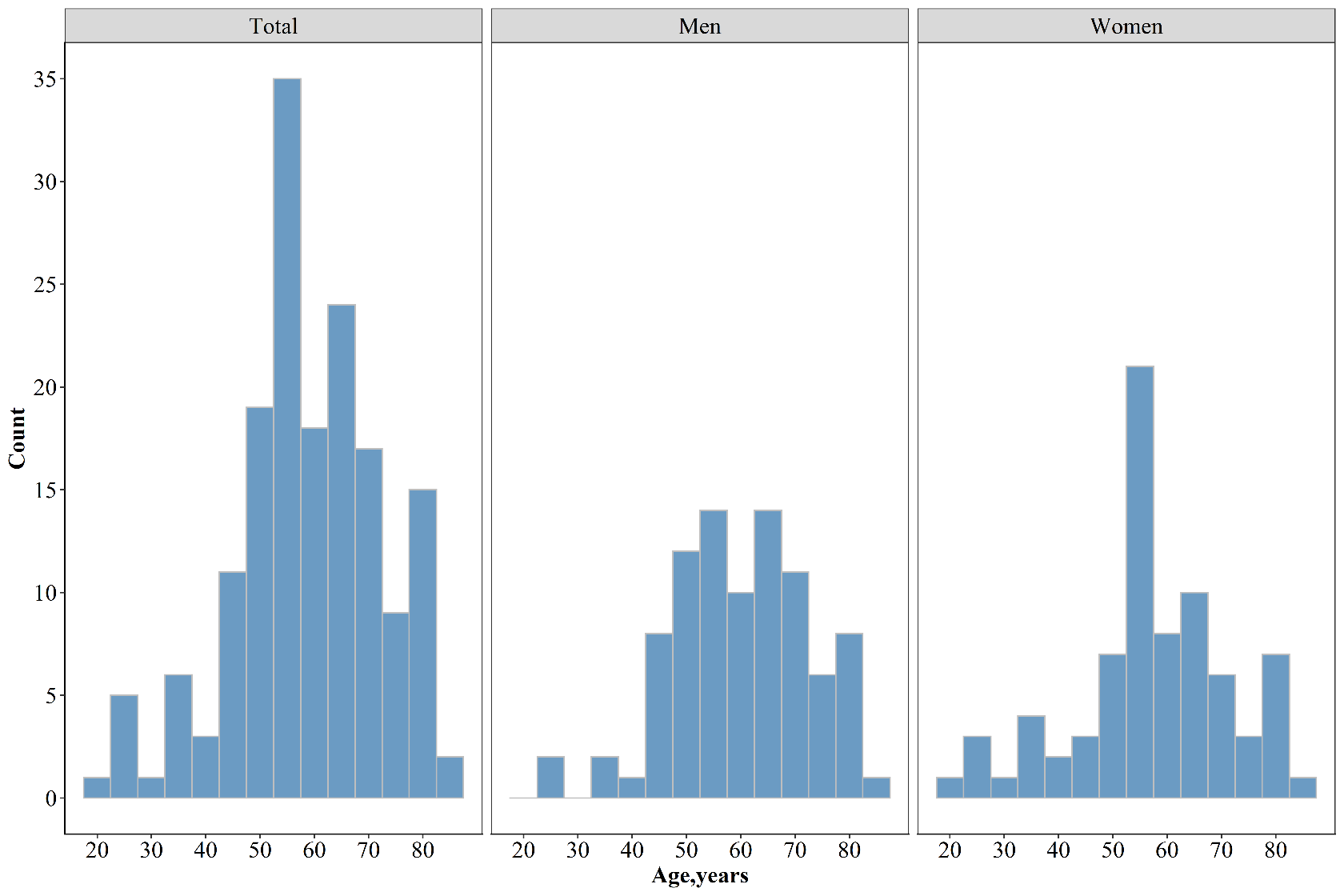
**

**Supplementary figure 2.** The age distribution of the study participants according to gender.

**Supplementary table 1.** Comparison of the characteristics of participants in this study with those in Henan rural cohort study.

| **Characteristics** | **Participants in this study (n=166)** | **Participants in Henan rural cohort (n=39259)** | ***P*** |
| --- | --- | --- | --- |
| Age (year), (mean and SD) | 58.80 (13.39) | 55.60 (12.19) | 0.001 |
| Gender n (%) |  |  |  |
| Men | 89 (53.61) | 15490 (39.46) | <0.001 |
| Women | 77 (46.39) | 23769 (60.54) |  |
| Marital status n (%) |  |  |  |
| Married/cohabiting | 149 (89.76) | 35243 (89.77) | 0.996 |
| Widowed/separated/divorced | 17 (10.24) | 4016 (10.23) |  |
| Educational level n (%) |  |  |  |
| Elementary school or below | 61 (36.75) | 17572 (44.76) | 0.112 |
| Junior high school | 77 (46.39) | 15643 (39.85) |  |
| Senior high school or above | 28 (16.87) | 6044 (15.40) |  |
| Average monthly income n (%) |  |  |  |
| <500 RMB | 35 (21.08) | 14014 (35.70) | <0.001 |
| 500- RMB | 45 (27.11) | 12907 (32.88) |  |
| ≥1000 RMB | 86 (51.81) | 12338 (31.43) |  |
| Smoking status n (%) |  |  |  |
| Current smoking | 45 (27.11) | 7487 (19.07) | 0.003 |
| Former smoking | 20 (12.05) | 3192 (8.13) |  |
| Never smoking | 101 (60.84) | 28580 (72.80) |  |
| Drinking status n (%) |  |  |  |
| Current drinking | 20 (12.05) | 7080 (18.03) | <0.001 |
| Former drinking | 37 (22.29) | 1832 (4.67) |  |
| Never drinking | 109 (65.66) | 30347 (77.30) |  |

Abbreviation: SD, standard deviation; RMB, Renminbi.

T-test was performed to compare the differences in continuous variables; Chi-square test was used to compare the differences in the categorical variables.

**Supplementary table 2.** The correlation coefficients of twice recall of body shape according to gender.

| **Age** | **N ^#^** | **SCC** | **PCC ^a^** | **PCC ^b^** | **ICC** |
| --- | --- | --- | --- | --- | --- |
| **Men** |  |  |  |  |  |
| 5 | 37 | 0.376^*^ | 0.563^**^ | 0.540^*^ | 0.549^**^ |
| 10 | 51 | 0.386^**^ | 0.434^*^ | 0.413^*^ | 0.411^*^ |
| 20 | 80 | 0.384^**^ | 0.429^**^ | 0.372^*^ | 0.427^**^ |
| 30 | 78 | 0.508^**^ | 0.557^**^ | 0.514^**^ | 0.552^**^ |
| 40 | 76 | 0.533^**^ | 0.577^**^ | 0.470^**^ | 0.577^**^ |
| 50 | 65 | 0.464^**^ | 0.452^**^ | 0.366^*^ | 0.451^**^ |
| 60 | 39 | 0.668^**^ | 0.610^**^ | 0.526^*^ | 0.596^**^ |
| 70 | 20 | 0.439 | 0.547^*^ | 0.638^*^ | 0.534^*^ |
| Now | 76 | 0.581^**^ | 0.501^**^ |  | 0.501^**^ |
| **Women** |  |  |  |  |  |
| 5 | 27 | 0.557^*^ | 0.559* | 0.547^*^ | 0.557^*^ |
| 10 | 40 | 0.469^*^ | 0.539** | 0.437^*^ | 0.536^**^ |
| 20 | 75 | 0.362^*^ | 0.388* | 0.386^*^ | 0.387^**^ |
| 30 | 70 | 0.338^*^ | 0.386** | 0.326^*^ | 0.380^*^ |
| 40 | 66 | 0.504^**^ | 0.474** | 0.414^*^ | 0.461^**^ |
| 50 | 53 | 0.344^*^ | 0.277* | 0.160 | 0.277^*^ |
| 60 | 23 | 0.463^*^ | 0.523* | 0.348 | 0.503^*^ |
| 70 | 13 | 0.588^*^ | 0.719* | 0.317 | 0.680^*^ |
| Now | 72 | 0.528^**^ | 0.466** |  | 0.466^**^ |

Abbreviation: SCC, Spearmen correlation coefficient; PCC ^a^, Pearson correlation coefficient; PCC ^b^, Partial correlation coefficient; ICC, Intraclass correlation coefficient.

**^#^** Sample sizes vary due to miss value.

^*^ *P* <0.05, ^**^ *P* <0.001.

**Supplementary table 3.** The correlation coefficients between first recall of body shape and BMI and WC according to gender.

| **Age group** | **BMI (kg/m^2^)/Weight (kg)** | |  | **WC (cm)** | | **SCC^†^** | **SCC^‡^** | **PCC^b†^** | **PCC^b‡^** |
| --- | --- | --- | --- | --- | --- | --- | --- | --- | --- |
|  | **n^#^** | **mean (SD)** |  | **n^#^** | **mean (SD)** |  |  |  |  |
| **Men** |  |  |  |  |  |  |  |  |  |
| Age 05 ^a^ | 57 | 23.24 (8.09) |  |  |  | 0.242 |  | 0.279^*^ |  |
| Age 10 | 70 | 25.46 (8.44) |  | 24 | 64.58 (9.33) | 0.251^*^ | 0.300 | 0.271^*^ | 0.384 |
| Age 20 | 89 | 21.74 (2.63) |  | 86 | 81.51 (8.35) | 0.640^**^ | 0.346^*^ | 0.587^**^ | 0.372^*^ |
| Age 30 | 86 | 22.41 (1.73) |  | 85 | 84.48 (10.03) | 0.636^**^ | 0.496^**^ | 0.659^**^ | 0.484^**^ |
| Age 40 | 82 | 22.94 (1.95) |  | 81 | 86.87 (10.92) | 0.734^**^ | 0.653^**^ | 0.720^**^ | 0.591^**^ |
| Age 50 | 69 | 23.65 (2.79) |  | 67 | 87.27 (10.08) | 0.549^**^ | 0.601^**^ | 0.255^*^ | 0.442^**^ |
| Age 60 | 43 | 23.66 (3.25) |  | 44 | 87.01 (11.47) | 0.599^**^ | 0.680^**^ | 0.390^*^ | 0.490^*^ |
| Age 70 | 23 | 22.86 (3.41) |  | 23 | 85.32 (9.99) | 0.424^*^ | 0.529^**^ | 0.185 | 0.214 |
| Now | 86 | 22.61 (3.44) |  | 86 | 87.70 (9.23) | 0.659^**^ | 0.606^**^ |  |  |
| **Women** |  |  |  |  |  |  |  |  |  |
| Age 05 ^a^ | 31 | 21.00 (5.99) |  |  |  | 0.331 |  | 0.259 |  |
| Age 10 | 48 | 19.79 (5.80) |  | 21 | 59.13 (4.84) | 0.209 | 0.360 | 0.349^*^ | 0.051 |
| Age 20 | 77 | 21.30 (3.44) |  | 75 | 69.81 (6.79) | 0.655^**^ | 0.515^**^ | 0.648^**^ | 0.507^**^ |
| Age 30 | 72 | 23.60 (3.61) |  | 72 | 75.10 (8.22) | 0.674^**^ | 0.670^**^ | 0.579^**^ | 0.567^**^ |
| Age 40 | 66 | 23.86 (3.86) |  | 66 | 78.21 (9.02) | 0.650^**^ | 0.594^**^ | 0.377^*^ | 0.437^**^ |
| Age 50 | 51 | 22.14 (3.05) |  | 48 | 81.67 (9.96) | 0.587^**^ | 0.432^*^ | 0.502^**^ | 0.296^*^ |
| Age 60 | 24 | 24.40 (3.69) |  | 24 | 80.72 (12.94) | 0.744^**^ | 0.661^**^ | 0.424^*^ | 0.304 |
| Age 70 | 13 | 25.64 (3.45) |  | 13 | 82.60 (12.33) | 0.850^**^ | 0.765^**^ | 0.190 | 0.722^*^ |
| Now | 74 | 25.35 (3.44) |  | 74 | 83.98 (9.47) | 0.689^**^ | 0.642^**^ |  |  |

Abbreviation: BMI, body mass index; WC, waist circumference; SD, standard deviation; SCC, spearmen correlation coefficient; PCC ^b^, partial correlation coefficient.

^a^ At age 05, weight (kg) was used to calculate the correlation coefficient.

^#^Sample sizes vary due to miss value.

**^†^**Body shape and BMI, **^‡^**Body shape and WC.

^*^*P* <0.05, ^**^*P* <0.001.

**Supplementary table 4.** The best cut-off value to define obesity based on recalled body shape, and the sensitivity and specificity of the World Health Organization’s (WHO) definition

|  | **Cutoff value** | **Sensitivity (%)** | **Specificity (%)** | **AUC (95% CI)** | ***P*** |
| --- | --- | --- | --- | --- | --- |
| **BMI** |  |  |  |  |  |
| Age group |  |  |  |  |  |
| Age 05 ^a^ | Body shape ≥ 2 | 79.41 | 43.75 | 0.595 (0.425, 0.764) | 0.241 |
| Age 10 ^a^ | Body shape ≥ 4 | 46.34 | 83.12 | 0.677 (0.569, 0.786) | 0.002 |
| Age 20 | Body shape ≥ 5 | 100.00 | 81.71 | 0.939 (0.843, 1.000) | 0.001 |
| Age 30 | Body shape ≥ 8 | 100.00 | 100.00 | 1.000 (1.000, 1.000) | <0.001 |
| Age 40 | Body shape ≥ 6 | 90.00 | 78.30 | 0.921 (0.846, 0.995) | <0.001 |
| Age 50 | Body shape ≥ 7 | 100.00 | 91.53 | 0.941 (0.895, 0.986) | <0.001 |
| Age 60 | Body shape ≥ 7 | 100.00 | 95.38 | 0.981 (0.946, 1.000) | <0.001 |
| Age 70 | Body shape ≥ 7 | 100.00 | 97.14 | 0.971 (0.916, 1.000) | <0.001 |
| Now | Body shape ≥ 7 | 100.00 | 93.51 | 0.973 (0.947, 0.999) | <0.001 |
| **WC** |  |  |  |  |  |
| Age group |  |  |  |  |  |
| Age 05 |  |  |  |  |  |
| Age 10 |  |  |  |  |  |
| Age 20 | Body shape ≥ 5 | 100.00 | 81.76 | 0.939 (0.842, 1.000) | 0.033 |
| Age 30 | Body shape ≥ 5 | 84.62 | 65.97 | 0.822 (0.719, 0.924) | <0.001 |
| Age 40 | Body shape ≥ 5 | 94.44 | 52.71 | 0.792 (0.698, 0.887) | <0.001 |
| Age 50 | Body shape ≥ 6 | 63.16 | 81.25 | 0.771 (0.638, 0.904) | <0.001 |
| Age 60 | Body shape ≥ 6 | 71.43 | 85.19 | 0.876 (0.781, 0.970) | <0.001 |
| Age 70 | Body shape ≥ 6 | 33.33 | 83.33 | 0.681 (0.462, 0.899) | 0.168 |
| Now | Body shape ≥ 6 | 65.71 | 80.00 | 0.774 (0.677, 0.871) | <0.001 |

Abbreviation: BMI, body mass index; WC, waist circumference; AUC, area under the curve; CI, confidence interval.

^a^ Gold standard of definition of obesity was that BMI-for-age greater than 2 standard deviations above the WHO Growth Reference median.
